# Supplementary material for: Ammonia Stress Induces Transcriptional Expression Changes in the Mature Eggs of the Acipenser baerii
Source: Animals (Basel). 2025 Oct 28;15(21):3122. doi: 10.3390/ani15213122 (PMC12609592; doi:10.3390/ani15213122)
Supplement: Supplementary file 1 [file animals-15-03122-s001.zip › animals-3890149-supplementary.pdf]

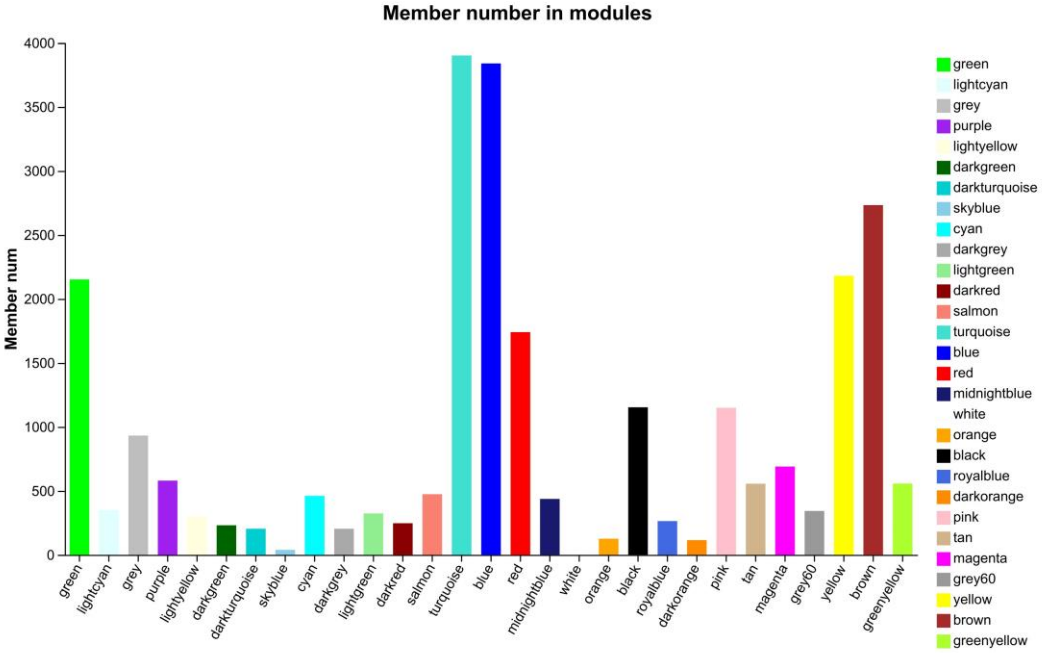


Figure S1. Statistical chart of the number of gene members contained in each module. The horizontal axis represents the module (indicated by module color); the vertical axis represents the number of members belonging to the module.

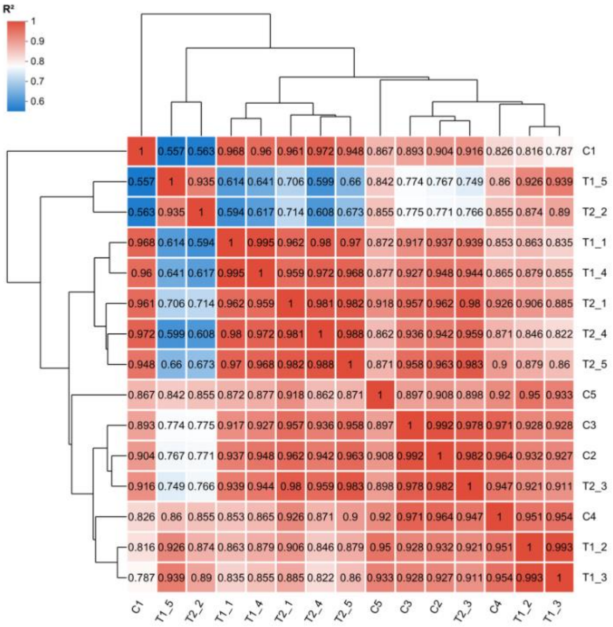


Figure S2. Correlation coefficient heatmap. On the right and bottom sides of the figure are the

sample names, while on the left and top sides are the clustering results of the samples. Different colors represent the magnitude of correlation coefficients between the samples.

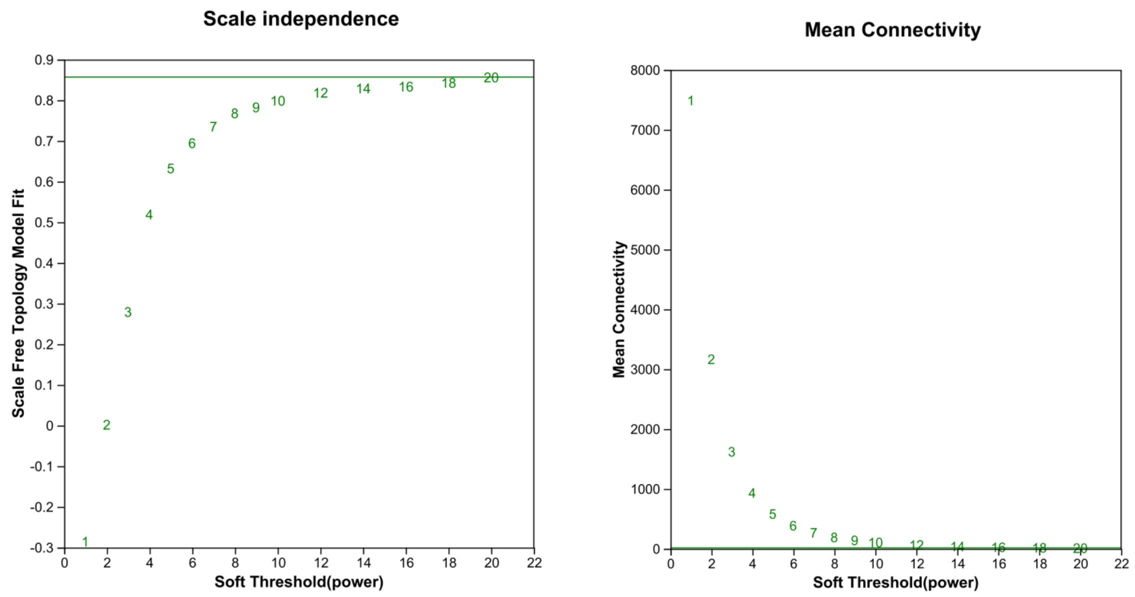


Figure S3. Analysis of network topology for various soft-thresholding powers. The left panel displays the scale-free fit index (y-axis) as a function of the soft-thresholding power (x-axis). The right panel shows the mean connectivity (degree, y-axis) as a function of the soft-thresholding power (x-axis).

Table S1. Primers for RT-qPCR validation

| | Primers | Forward sequences | Reverse sequences | Length (bp) |
|---|---|---|---|---|
| TRINITY_DN15088_c0_g1 | *GCLM* | AGCTGCTGACCCACAATGAT | CCCACTCCAGTGACCAATCC | 111 |
| TRINITY_DN8805_c0_g1 | *GST* | GCTGGCGTTGAGTTTGAGG | TTCAGGTCCTTCCCATAGAGG | 185 |
| TRINITY_DN22067_c0_g1 | *CPY17* | AGGACGCTTCGAGAGGAGAT | GCAGCCGCTGAAAGAGAATG | 125 |
| TRINITY_DN53652_c0_g1 | *CPY11A1* | CATCAGGGACTCTGGTGCAG | GGGACCGAACCCAAAGCTAA | 143 |
| TRINITY_DN25220_c0_g1 | *CPY19A1* | TGATCTGTCTCAGCACCCCA | CTATACAGGACCGAGGGCCA | 128 |
| TRINITY_DN11431_c0_g2 | *3βHSD* | ACGGAATCAGAAACGGGGAC | CCCTCATAGCTTTGGCTGCT | 117 |
| Reference gene | *β-actin* | TGGACGCCCAAGACATCAGG | TCTCCATGGTGGTGAACACG | 105 |
